# Impact of contacting study authors to obtain additional data for systematic reviews: diagnostic accuracy studies for hepatic fibrosis

**DOI:** 10.1186/2046-4053-3-107

**Published:** 2014-09-19

**Authors:** Shelley S Selph, Alexander D Ginsburg, Roger Chou

**Affiliations:** 1 The Pacific Northwest Evidence-based Practice Center, Department of Medical Informatics and Clinical Epidemiology, Oregon Health & Science University, 3181 SW Sam Jackson Park Road, Mail Code: BICC, Portland, OR 97239, USA; 2 Department of Medicine, Oregon Health & Science University, Portland, OR, USA

**Keywords:** Contacting study authors, Diagnosing hepatic fibrosis, Hepatitis C infection, Systematic review

## Abstract

**Background:**

Seventeen of 172 included studies in a recent systematic review of blood tests for hepatic fibrosis or cirrhosis reported diagnostic accuracy results discordant from 2 × 2 tables, and 60 studies reported inadequate data to construct 2 × 2 tables. This study explores the yield of contacting authors of diagnostic accuracy studies and impact on the systematic review findings.

**Methods:**

Sixty-six corresponding authors were sent letters requesting additional information or clarification of data from 77 studies. Data received from the authors were synthesized with data included in the previous review, and diagnostic accuracy sensitivities, specificities, and positive and likelihood ratios were recalculated.

**Results:**

Of the 66 authors, 68% were successfully contacted and 42% provided additional data for 29 out of 77 studies (38%). All authors who provided data at all did so by the third emailed request (ten authors provided data after one request). Authors of more recent studies were more likely to be located and provide data compared to authors of older studies. The effects of requests for additional data on the conclusions regarding the utility of blood tests to identify patients with clinically significant fibrosis or cirrhosis were generally small for ten out of 12 tests. Additional data resulted in reclassification (using median likelihood ratio estimates) from less useful to moderately useful or vice versa for the remaining two blood tests and enabled the calculation of an estimate for a third blood test for which previously the data had been insufficient to do so. We did not identify a clear pattern for the directional impact of additional data on estimates of diagnostic accuracy.

**Conclusions:**

We successfully contacted and received results from 42% of authors who provided data for 38% of included studies. Contacting authors of studies evaluating the diagnostic accuracy of serum biomarkers for hepatic fibrosis and cirrhosis in hepatitis C patients impacted conclusions regarding diagnostic utility for two blood tests and enabled the calculation of an estimate for a third blood test. Despite relatively extensive efforts, we were unable to obtain data to resolve discrepancies or complete 2 × 2 tables for 62% of studies.

## Background

Systematic reviewers often identify studies containing discordant, inconsistent, or missing data. Studies with such deficiencies can potentially influence the outcome of quantitative and qualitative synthesis of results. As a result, determining the best strategy to address incomplete, inaccurate, or missing data is a major methodological challenge in conducting systematic reviews.

The problem of missing data in systematic reviews appears to be common. A 2006 meta-analysis of weight loss interventions found that 40% of 604 studies had missing or incomplete data on important variables such as age and sample size [1]. Similarly, a 2004 review of the effects of aerobic exercise on lipids and lipoproteins found that 22% of 174 studies had missing data [2].

One suggested strategy for addressing this issue is for systematic reviewers to contact study authors to clarify discordant data or to obtain missing data [3,4]. However, there is little known about the yield of requests for data or the effects of data obtained through author contact on the findings of systematic reviews. A 2009 review found that 50% of 93 systematic reviews in the 25 medical journals with the highest impact factors and 85% of 54 Cochrane systematic reviews published between 2005 and 2006 report contacting authors [5]. Further, 43% of reviews in the top medical journals and 83% of Cochrane reviews describe the process of author contact. However, only 4% of journal reviews and 9% of Cochrane reviews reported the response rates to author contacts. Evidence regarding the yield and impact of author requests is particularly sparse in the area of diagnostic tests.

In 2012, the Pacific Northwest Evidence-based Practice Center conducted a systematic review to determine the diagnostic accuracy of various blood tests for hepatic fibrosis or cirrhosis in patients with chronic hepatitis C viral infection [6-8]. We found evidence that a number of blood tests are useful for identifying clinically significant fibrosis or cirrhosis, based on positive likelihood ratios of 5 to 10, suggesting a potential role as an alternative to liver biopsy. However, of the 172 included studies, 17 studies reported data that were discordant from 2 × 2 tables (i.e., number of true positives, false positives, true negatives, and false negatives) calculated from the information provided (e.g., prevalence of fibrosis or cirrhosis, sensitivity, and specificity) in the studies. In addition, 60 studies were missing necessary data for one or more diagnostic tests to be included in summary estimates. To the authors’ knowledge, this is the first study to evaluate the responsiveness of authors contacted to clarify discordant data or obtain missing data and the impact of the additional data provided in studies of diagnostic accuracy.

## Methods

### Included studies

Based on the previous systematic review [6-8], we identified 17 studies [9-25] that had discrepancies in the data reported and 60 studies [26-86] that provided insufficient data to construct 2 × 2 tables at standard cutoffs for one or more diagnostic tests. We defined studies with discrepancies as those in which reported measures of diagnostic accuracy were inconsistent with measures of diagnostic accuracy calculated from 2 × 2 tables by values of >0.10 (e.g., reported a positive predictive value of 0.85 vs. calculated a positive predictive value of 0.70). For studies in which 2 × 2 table data were not provided, we calculated values for 2 × 2 tables for commonly reported cutoff values for a positive test, based on the reported sample size, prevalence of the condition of interest (fibrosis or cirrhosis), sensitivity, and specificity. Studies for which we could not construct 2 × 2 tables included those in which some measures of diagnostic accuracy were reported, but other necessary information was missing (e.g., sample size, prevalence of condition); studies in which sensitivity and specificity were reported at non-standard cutoffs; and studies in which an area under the receiver operating characteristic (AUROC) was reported without sensitivity or specificity at standard cutoffs.

### Contacting authors

We requested data from 66 corresponding authors from around the world (Table 1) for 77 studies. All publications were in English and all corresponding authors were contacted in English. We sent corresponding authors an initial request for additional data by email. For the convenience of authors, we provided labeled 2 × 2 tables they could fill in and send back to us. If there was no response to our initial email, after a minimum of three business days, we sent a second reminder email to the corresponding author. If there was still no response after a minimum of eight business days following the initial email, we sent a second reminder email. After a minimum of ten business days with no response, we then attempted to contact authors by telephone. If still unable to reach corresponding authors, we attempted to contact the last authors and statisticians, if identifiable. If corresponding authors forwarded our request to other authors, we sent reminders to these authors. After a minimum of 15 business days from our initial email, we sent a final email to authors. If we received an automated “out-of-office” response, we waited until the author had returned to send further reminders.

**Table 1:** Number of authors contacted and provided data by country

**Country**	**Authors contacted**	**Provided data**
Austria	1	0
Belgium	2	1
Brazil	3	1
Egypt	7	2
France	12	5
Germany	2	1
Israel	2	1
Italy	6	3
Japan	2	1
Korea	2	2
Luxembourg	1	1
Mexico	1	0
The Netherlands	1	1
Pakistan	1	0
Romania	4	3
Spain	1	0
Sweden	1	0
Taiwan	2	1
Tunisia	1	0
Turkey	2	1
United Kingdom	5	0
United States	7	4
Total	66	28

### Incorporation of data

For studies with discrepancies and cases in which we could not construct a 2 × 2 table, we requested that authors provide the 2 × 2 data used to generate their estimates of diagnostic accuracy. For studies that provided only AUROC or did not report diagnostic accuracy at standard cutoffs, we asked that authors provide 2 × 2 data for diagnostic accuracy at standard cutoffs for the blood test or tests evaluated.

We recalculated median values and ranges for sensitivity and specificity at the cutoffs used in the original review using additional data obtained, and we compared differences between the updated and original findings. We categorized blood tests reporting a positive likelihood ratio of 5 to 10 or a negative likelihood ratio of 0.1 to 0.2 as moderately useful (no blood test was associated with a positive likelihood ratio of >10 or negative likelihood ratio <0.1) [87]. We also reassessed the strength of evidence with the additional data.

We compared the recalculated sensitivity, specificity, positive likelihood ratio, and negative likelihood ratio to the pooled estimates from the initial review. In addition, we compared the new strength of evidence ratings to that based on the dataset from the initial review.

## Results

### Response rate

**Figure 1:**
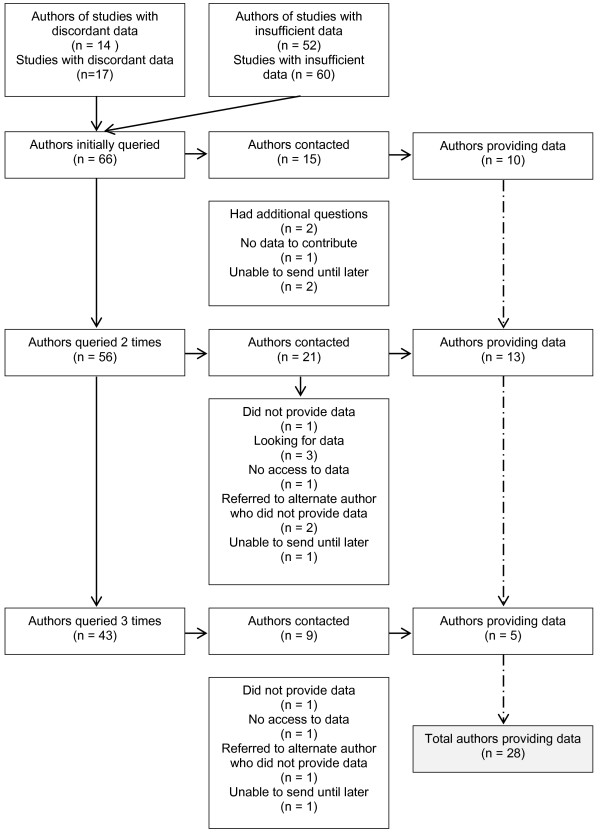
Study authors and results of contact attempts.

Of the 66 authors, we were able to contact 45 (68%) (Figure 1). Of those 45 authors, 28 provided additional data for 29 studies, including four who provided datasets. Among authors whom we were able to contact, reasons for not sending data included the following: no current access to the data and need for additional time to find and format the data (e.g., data stored on a floppy disk).

All authors who provided data did so by the third request for information (second reminder). We received information from ten authors after only one request. Two requests were required for 13 authors, and three were required for five authors. The average number of total days between the initial request and the first reminder was seven, between the first reminder and the second was 13, and between the second reminder and the third was 16. The minimum and maximum number of days between any two contact attempts was three and 34, respectively. Several authors were on holiday or sabbatical, and we waited until their return to continue sending reminders which resulted in longer times between requests. We received no additional information after three requests and received no additional data in response to telephone contact.

There was no difference in the likelihood of providing data between authors of studies with discrepancies compared with authors of studies in which 2 × 2 tables could not be calculated (54% vs. 40%, *p* = 0.36). Of the 17 studies in which there was a discrepancy between reported results for diagnostic accuracy and constructed 2 × 2 tables, seven of 13 authors provided data on seven studies [9,12,13,16,18,20,25], including one dataset [18]. We were unable to contact four authors [11,14,19,21], one author forwarded our request to a colleague who did not provide the data [22-24], one provided data for one of two studies [17], and one declined telephone contact [15]. Of the 60 studies missing information to generate 2 × 2 tables, 21 of 53 authors provided additional or confirmatory data on 22 studies [28,30-35,39,49,51,52,57,62,66,68,69,71,77,78,81,85,86], including three datasets [49,77,78]. Reasons for not providing data were similar to those for authors of studies with discrepancies. Authors of more recent studies were more likely to be located and provide data (*p* = 0.02). The mean year of publication of studies for which we received additional data was 2010. The mean year of publication of studies by contacted authors who did not provide additional data was 2008, while the average publication year for authors of studies we could not locate was 2007. Country of publication did not appear to predict the likelihood of receiving data (Table 1).

### Effect on diagnostic accuracy

For diagnosing hepatic fibrosis, additional data were provided for 12 out of 16 blood tests. The number of additional studies for specific tests and cutoffs ranged from zero to nine (zero additional studies occurred when additional data were obtained, but only for studies with discrepancies, so that one set of data was replaced by another) (Tables 2 and 3) There was little impact on median estimates of diagnostic accuracy for the two tests with the greatest number of additional studies added (five and ten studies). See the full report for specific tests affected [88].

**Table 2:** Diagnostic accuracy of tests for fibrosis

**Fibrosis test (cutoff)**	**Number of samples**	**Sensitivity (median, range)**	**Specificity (median, range)**	**Positive likelihood ratio (median, range)**	**Negative likelihood ratio (median, range)**
Platelets <140 to <163	8	0.56 (0.28–0.89)	0.91 (0.69–1.0)	6.3 (2.3–14)	0.48 (0.16–0.78)
With additional data	10^a^	0.57 (0.28–0.89)	0.91 (0.58–1.0)	6.3 (1.64–35)	0.48 (0.16–0.78)
API >3.5 or ≥4.0	5	0.70 (0.52–0.82)	0.70 (0.51–0.77)	2.3 (1.7–2.7)	0.43 (0.34–0.67)
With additional data	6^a^	0.64 (0.52–0.82)	0.69 (0.51–0.77)	2.0 (1.7–2.7)	0.53 (0.34–0.67)
API ≥6.0	5/3^b^	0.51 (0.19–0.75)	0.90 (0.58–0.96)	*5.1 (1.8* ***–*** *7.3)*	0.54 (0.43–0.94)
With additional data	6^a^	0.54 (0.19–0.75)	0.88 (0.58–0.96)	*4.5 (1.4* ***–*** *7.3)*	0.52 (0.43–0.94)
APRI ≥0.5 to >0.55	28	0.81 (0.29–0.98)	0.55 (0.10–0.94)	1.8 (1.1–4.8)	0.35 (0.08–0.78)
With additional data	40^c^	0.79 (0.29–0.98)	0.56 (0.10–1.0)	1.8 (1.0–7.5)	0.56 (0.07–0.93)
AST: ALT ratio >1.0	5	0.35 (0.08–0.45)	0.77 (0.62–1.0)	1.5 (1.1–15)	0.84 (0.84–0.98)
With additional data	8^a,c^	0.36 (0.08–0.59)	0.80 (0.48–1.0)	1.7 (1.1–14)	0.81 (0.65–0.98)
ELF >8.75, >9.0, or >9.78	3	0.85 (0.84–0.86)	0.70 (0.62–0.80)	2.8 (2.3–4.2)	0.21 (0.19–0.23)
With additional data	3^a^	0.84 (0.62–0.85)	0.80 (0.70–0.86)	4.2 (2.8–4.4)	0.20 (0.19–0.45)
FIB-4 > 1.26 or ≥1.45	6	0.64 (0.62–0.86)	0.68 (0.54–0.75)	2.0 (0.88–2.6)	0.53 (0.21–1.3)
With additional data	9^c^	0.64 (0.57–0.86)	0.68 (0.28–0.85)	2.0 (0.88–3.7)	0.53 (0.21–1.3)
FIB-4 > 3.25	4	0.50 (0.28–0.86)	0.79 (0.59–0.99)	*2.4 (1.3* ***–*** *28)*	0.63 (0.21–0.80)
With additional data	7^c^	0.28 (0.11–0.86)	0.97 (0.59–1.0)	*9.3 (1.3* ***–*** *28)*	0.74 (0.21–0.89)
FibroIndex >1.25	3	0.94 (0.62–0.97)	0.40 (0.40–0.48)	1.6 (1.2–1.6)	*0.15 (0.08* ***–*** *0.79)*
With additional data	6^c^	0.64 (0.54–0.97)	0.57 (0.40–1.0)	1.5 (1.2–2.2)	*0.62 (0.08* ***–*** *0.79)*
FibroIndex >2.25 or ≥2.25	3	0.30 (0.17–0.36)	0.97 (0.97–1.0)	10,12,∞	0.72 (0.66–0.83)
With additional data	4^c^	0.24 (0.14–0.36)	0.99 (0.97–1.0)	10,12,∞	0.78 (0.66–0.87)
FibroMeter >0.419 to >0.59	3	0.69 (0.64–0.80)	0.81 (0.76–0.81)	3.6 (3.4–3.6)	0.38 (0.26–0.44)
With additional data	5^c^	0.80 (0.64–0.87)	0.76 (0.64–0.81)	3.3 (2.4–3.6)	0.26 (0.21–0.44)
FibroTest >0.10 to >0.22	6	0.92 (0.88–0.97)	0.38 (0.27–0.56)	1.5 (1.3–1.9)	*0.21 (0.11* ***–*** *0.28)*
With additional data	9^c^	0.92 (0.64–0.98)	0.46 (0.21–1.0)	1.7 (1.2–2.2)	*0.17 (0.11* ***–*** *0.39)*
FibroTest >0.70 or >0.80	5	0.22 (0.20–0.50)	0.96 (0.95–0.98)	5.5 (5.5–13)	0.81 (0.53–0.82)
With additional data	10^a,c^	0.38 (0.20–0.94)	0.95 (0.36–0.98)	7.6 (1.4–13)	0.65 (0.12–0.82)
Forns Index >4.2 to >4.57	14	0.88 (0.57–0.94)	0.52 (0.20–0.77)	1.8 (1.2–2.2)	0.22 (0.12–0.64)
With additional data	16^a,c^	0.89 (0.42–0.94)	0.51 (0.20–0.77)	1.8 (0.54–2.2)	0.23 (0.12–2.6)
Forns Index >6.9	10	0.36 (0.18–0.61)	0.94 (0.66–1.0)	6.5 (1.6–18)	0.68 (0.56–0.92)
With additional data	14^c^	0.40 (0.18–0.81)	0.95 (0.33–1.0)	7.4 (1.2–18)	0.63 (0.22–0.92)
Hepascore >0.46 to ≥0.55	5	0.66 (0.54–0.82)	0.79 (0.65–0.86)	3.1 (2.3–4.5)	0.43 (0.28–0.55)
With additional data	8^c^	0.65 (0.54–0.82)	0.80 (0.65–0.86)	3.2 (2.3–4.5)	0.44 (0.28–0.55)
Lok Index >0.17 or >0.20	0	NA	NA	NA	NA
With additional data	3^c^	0.58 (0.48–0.82)	0.80 (0.58–0.81)	2.9 (2.0–3.1)	0.53 (0.31–0.65)

Values in italics indicate a change to above or below a cutoff of 5.0 for positive likelihood ratio or 0.20 for negative likelihood ratio.

*ALT* serum alanine aminotransferase, *API* age platelet index, *APRI* aspartate aminotransferase to platelet ratio index, *AST* aspartate aminotransferase, *ELF* enhanced liver fibrosis, *NA* not available.

^a^Additional data for study(s) with discrepancy in reported data.

^b^The first number is the number of samples for sensitivity/the second number is the number of samples for specificity.

^c^Additional data for study(s) without 2 × 2 tables.

**Table 3:** Diagnostic accuracy of tests for cirrhosis

**Fibrosis test (cutoff)**	**Number of samples**	**Sensitivity (median, range)**	**Specificity (median, range)**	**Positive likelihood ratio (median, range)**	**Negative likelihood ratio (median, range)**
Platelets <140 to <155	9	0.78 (0.41–0.93)	0.87 (0.84–0.94)	6.0 (2.8–93)	0.25 (0.07–0.63)
With additional data	10^a^	0.77 (0.41–0.93)	0.86 (0.57–0.99)	5.5 (1.6–93)	0.27 (0.07–0.63)
API ≥6.0	5/3^b^	0.67 (0.43–0.80)	0.87 (0.81–0.93)	5.2 (2.7–10)	0.38 (0.22–0.68)
With additional data	6^a^	0.64 (0.12–0.80)	0.88 (0.81–0.99)	5.3 (2.7–17)	0.41 (0.22–0.88)
APRI >1.0 or ≥1.0	19	0.77 (0.33–1.0)	0.75 (0.30–0.87)	3.1 (1.4–4.9)	0.31 (0–0.77)
With additional data	30/29^b,c^	0.75 (0.13–1.0)	0.77 (0.30–1.0)	3.2 (1.4–10.6)	0.33 (0–0.89)
AST: ALT ratio >1.0	17	0.36 (0.12–0.78)	0.92 (0.59–1.0)	*4.5 (1.0* ***–*** *31)*	0.70 (0.47–1.0)
With additional data	19^a^	0.39 (0.10–0.78)	0.93 (0.59–1.0)	*5.6 (1.0* ***–*** *31)*	0.66 (0.23–1.0)
FIB-4 > 1.45	1	0.90	0.58	2.1	0.17
With additional data	4^c^	0.89 (0.87–1.0)	0.58 (0.40–0.70)	2.1 (1.7–2.9)	0.19 (0.0–0.23)
FIB-4 > 3.25	1	0.55	0.92	6.9	0.49
With additional data	5^c^	0.49 (0.40–0.55)	0.93 (0.91–0.95)	6.4 (5.7–8.9)	0.60 (0.49–0.63)
FibroTest >0.56 or >0.66	2	0.85 and 0.82	0.74 and 0.77	3.3 and 36	0.20 and 0.23
With additional data	7^c^	0.83 (0.27–0.91)	0.74 (0.65–1.0)	3.6 (2.6–3.6)	0.23 (0.11–0.73)
FibroTest >0.73, >0.75, >0.862	7	0.56 (0.30–1.0)	0.81 (0.24–0.96)	2.9 (1.2–10)	0.54 (0.0–0.79)
With additional data	10^a,c^	0.49 (0.11–0.86)	0.89 (0.55–1.0)	4.3 (1.2–11)	0.57 (0.20–0.89)
Forns Index >4.2	1	0.98	0.27	1.3	0.07
With additional data	6^c^	0.66 (0.27–1.0)	0.31 (0–1.0)	1.4 (0.27–1.5)	0.07 (0–0.66)
Forns Index >6.9	1	0.67	0.91	7.4	0.36
With additional data	3^c^	0.66 (0.53–0.67)	0.87 (0.86–0.91)	5.2 (4.2–7.4)	0.39 (0.36–0.53)
Lok Index ≥0.20 or >0.26	6	0.90 (0.67–1.0)	0.50 (0.30–0.82)	1.8 (1.0–4.8)	*0.21 (0–0.94)*
With additional data	7^c^	0.90 (0.67–1.0)	0.53 (0.30–0.82)	1.9 (1.0–4.8)	*0.19 (0–0.94)*
Lok Index ≥0.5 or >0.6	7	0.53 (0.40–0.79)	0.88 (0.60–0.95)	*4.4 (1.3* ***–*** *11)*	0.53 (0.24–0.80)
With additional data	8^c^	0.53 (0.23–0.79)	0.91 (0.60–0.97)	*5.8 (1.3* ***–*** *11)*	0.52 (0.24–0.80)

Values in italics indicate a change to above or below a cutoff of 5.0 for positive likelihood ratio or 0.20 for negative likelihood ratio.

*ALT* serum alanine aminotransferase, *API* age platelet index, *APRI* aspartate aminotransferase to platelet ratio index, *AST* aspartate aminotransferase.

^a^Additional data for study(s) with discrepancy in reported data.

^b^The first number is the number of samples for sensitivity/the second number is the number of samples for specificity.

^c^Additional data for study(s) without 2 × 2 tables.

Additional data for two tests for fibrosis resulted in a meaningful change in test usefulness from less useful to moderately useful for one test and from moderately useful to less useful for one test. Although the additional data resulted in the reclassification of two additional blood tests, the actual change in median estimates was small to minimal. Additional data also enabled us to create estimates of diagnostic accuracy for fibrosis for one test, for which data had previously been insufficient to do so.

For diagnosing cirrhosis, additional data were provided for eight of 16 blood tests. For the test with the greatest number of additional studies (ten studies), the effect on median likelihood ratio estimates was minimal [88]. The number of additional studies ranged from one to five for other blood tests. Additional data for two tests enabled reclassification from less useful to moderately useful, but the impact on the actual estimates was minimal.

We compared the effects of additional data from studies with discrepancies with the effects of additional data from studies in which 2 × 2 tables could not be generated and found no clear pattern suggesting differential effects on median estimates. We also evaluated effects of additional data with respect to the original strength of evidence ratings. The overall strength of evidence rating did not change for any of the tests for which we obtained additional data. The test for which we received the most additional data was already rated high strength of evidence.

## Discussion

Our experience demonstrates that obtaining additional data through author contacts for studies of diagnostic accuracy is possible, although challenging. We were able to contact the majority of authors (45 out of 66). Most contacted authors (28 out of 45) provided data, and several more indicated that they would have had the data been more readily accessible to them. Although the effects of the additional data on summary estimates were relatively small in most cases, the changes had important implications in assessing the clinical utility of two tests, in one case moving a blood test into the moderately useful range and in the other case moving it out of the moderately useful range. This suggests that while including previously unpublished data can result in clinically important changes in estimates, the magnitude and direction of impact may not be readily predictable.

Although we successfully contacted 68% of authors, this effort was time consuming, not only for us but also for study authors, who often had to first locate the data before being able to complete the 2 × 2 tables. In addition, despite our efforts, data to resolve discrepancies or calculate 2 × 2 tables at commonly used cutoffs for sensitivity and specificity could not be obtained for 48 of 77 (62%) studies, most frequently because authors could not be contacted or because they did not have access to the data. This experience indicates that despite relatively extensive efforts to obtain additional data, unresolved discrepancies and missing data remain likely. All data were obtained with the first three out of five attempted contacts, suggesting that more extensive efforts may be of low yield. In particular, telephone contact did not produce any additional information.

### Limitations

Receiving data was a function of not only whether authors were accessible and willing to send data but also whether they were able to communicate in English. As a result, a slightly higher yield may have been possible if non-English-speaking authors had been contacted in their native language.

## Conclusions

Contacting authors of studies evaluating the diagnostic accuracy of serum biomarkers for hepatic fibrosis and xcirrhosis in hepatitis C patients to obtain additional data was successful for 29 of 77 studies (38%). This resulted in changes in estimates and reclassification of two tests for hepatic fibrosis and the inclusion of an additional test for which data had previously been insufficient to calculate an estimate. Systematic reviewers with adequate resources should consider contacting authors of studies with missing or discrepant data, especially if these studies were published within the past 4 years. However, despite relatively extensive efforts, we were unable to obtain data to resolve discrepancies or complete 2 × 2 tables for 48 of 77 studies. Given that three attempts were needed to obtain even that level of information, more efficient mechanisms of achieving better access to information are needed. Requiring authors of studies on diagnostic accuracy to provide the 2 × 2 tables at commonly used cutoffs in the original study publication (or in the results of publicly available trial registries such as ClinicalTrials.gov) or requiring authors to make their datasets publicly available would save time, enable systematic reviewers to synthesize data more readily and completely, and enable more transparent verification of authors’ estimates of diagnostic accuracy.

## Abbreviations

ALT: alanine aminotransferase; API: age platelet index; APRI: aspartate aminotransferase to platelet ratio index; AST: aspartate aminotransferase; AUROC: area under the receiver operating characteristic.

## Competing interests

The authors declare that they have no competing interests.

## Authors’ contributions

SS and AG analyzed the data and drafted the manuscript. RC conceived the study and revised the manuscript. All authors read and approved the final manuscript.
